# Supplementary material for: Understanding Micronutrient Access through the Lens of the Social Ecological Model: Exploring the Influence of Socioeconomic Factors—A Qualitative Exploration
Source: Nutrients. 2024 Jun 4;16(11):1757. doi: 10.3390/nu16111757 (PMC11174576; doi:10.3390/nu16111757)
Supplement: Supplementary file 1 [file nutrients-16-01757-s001.zip › nutrients-2956713-supplementary.pdf]

Table S1: Quotes Used to Make Themes

Theme 1: Individual-Level Factors		
**Participant**	**Code**	**Transcript Excerpt**
P1	Personal dietary habits	"I try to eat healthy most of the time, but sometimes convenience wins over nutrition."
P2	Nutritional awareness	"I've been trying to educate myself more about nutrition, but there's so much conflicting information."
P5	Food preferences	"I grew up eating certain foods, and it's hard to break those habits even when I know better."
P7	Health-conscious choices	"I'm always mindful of what I put into my body and try to make health-conscious choices."
P12	Eating patterns	"I tend to skip meals when I'm busy, and then I end up overeating later in the day."
P15	Dietary restrictions	"I have to avoid gluten, so that limits my food options quite a bit."
P20	Meal planning	"I try to plan my meals for the week ahead to stay on track with my nutrition goals."
P25	Cooking skills	"I'm not very confident in the kitchen, so I stick to simple recipes most of the time."
P30	Food budgeting	"I have to be careful with my food budget, so I look for affordable options when I shop."
P33	Shopping behaviors	"I try to shop the perimeter of the grocery store for fresh produce and avoid processed foods."
P4	Personal dietary habits	"I enjoy indulging in my favorite comfort foods occasionally, but I balance it with healthier choices."
P9	Nutritional awareness	"I've started reading food labels more carefully to watch out for hidden sugars and additives."

P14	Food preferences	"I have a sweet tooth, so I struggle to resist sugary snacks even though I know they're not good for me."
P18	Health-conscious choices	"After learning about the importance of hydration, I make sure to drink enough water throughout the day."
P22	Eating patterns	"I've noticed that I tend to eat when I'm stressed, so I'm working on finding healthier coping mechanisms."
P27	Dietary restrictions	"I'm lactose intolerant, so I have to avoid dairy products, which can be challenging at times."
P29	Meal planning	"I like to prepare big batches of meals in advance and freeze them for busy days."
P31	Cooking skills	"Cooking has become a hobby for me, and I enjoy experimenting with new recipes."
P35	Food budgeting	"I've found that buying in bulk helps me save money on groceries without sacrificing quality."
P8	Shopping behaviors	"I try to support local farmers by purchasing fresh produce from farmers' markets when I can."

Theme 2: Interpersonal Relationships

Participant	Code	Transcript Excerpt
P3	Family support	"My family always encourages me to eat well and stay healthy, which motivates me to make better food choices."
P6	Friend influence	"When I see my friends making healthy food choices, it inspires me to do the same."
P10	Social network connections	"Being part of a community of health-conscious individuals has made it easier for me to maintain my healthy habits."
P13	Family support	"My family and I cook meals together, which not only strengthens our bond but also ensures we eat nutritious foods."

P17	Friend influence	"I often dine out with friends, and their food choices sometimes influence what I order."
P21	Social network connections	"I've joined online forums where people share healthy recipes and tips, and it's been a great source of motivation."
P24	Family support	"My family has always emphasized the importance of eating balanced meals, so it's ingrained in my lifestyle."
P28	Friend influence	"My friends and I take turns hosting dinner parties where we cook healthy meals and share recipes."
P32	Social network connections	"I have a group of friends who are all into fitness, and we motivate each other to stick to our nutrition goals."
P34	Family support	"My family has been incredibly supportive of my dietary restrictions and always makes sure there's something for me to eat."
P1	Family support	"My family doesn't always understand my dietary choices, but they respect them and try to accommodate them."
P4	Friend influence	"I have a friend who's a nutritionist, and she's been a great source of guidance in making healthier food choices."
P9	Social network connections	"I've made friends at the gym who share similar dietary goals, and we often swap recipes and meal prep together."
P12	Family support	"My family has a tradition of cooking together on weekends, and it's something I look forward to every week."
P16	Friend influence	"My friends and I enjoy trying out new restaurants, but lately, we've been opting for places with healthier options."
P20	Social network connections	"I've joined a cooking club where we take turns hosting themed dinners, and it's been a fun way to explore new recipes."
P25	Family support	"My family has been my biggest cheerleaders in my weight loss journey, and their support keeps me motivated."

P30	Friend influence	"My friend is a personal trainer, and he's helped me understand the importance of nutrition in achieving my fitness goals."
P33	Social network connections	"I've connected with other moms in my neighborhood, and we organize healthy potlucks and share tips for feeding our families nutritious meals."
P35	Family support	"My family and I have a tradition of Sunday brunches, where we catch up and enjoy wholesome meals together."

Theme 3: Community Environment

Participant	Code	Transcript Excerpt
P2	Access to food stores	"Living in a neighborhood with no grocery stores nearby makes it challenging to access fresh produce regularly."
P5	Community programs	"Our community center offers cooking classes and nutrition workshops, which have been instrumental in improving my diet."
P8	Local policies	"I've noticed that the local government has been implementing policies to encourage healthier food options in schools."
P11	Neighborhood environments	"My neighborhood has several urban gardens, and being able to grow my own vegetables has made healthy eating more accessible."
P14	Access to food stores	"I rely on public transportation, and there aren't many grocery stores along the route, making it inconvenient to shop for fresh produce."
P18	Community programs	"The farmers' market in our neighborhood not only provides fresh produce but also hosts nutrition workshops and cooking demonstrations."
P22	Local policies	"I've noticed that there are more bike lanes and pedestrian-friendly streets in my community, which encourages outdoor activities and healthier lifestyles."

P26	Neighborhood environments	"My neighborhood has a strong sense of community, and we often organize potlucks and food swaps to share healthy recipes and ingredients."
P29	Access to food stores	"Living in a food desert has forced me to travel farther to find affordable groceries, and it's a challenge to maintain a healthy diet."
P31	Community programs	"Our local community center offers subsidized cooking classes for low-income families, which have been a lifeline for me in learning how to prepare nutritious meals on a budget."
P35	Local policies	"Our city council recently passed a resolution to ban fast-food advertisements near schools, which I believe will positively impact children's dietary habits."

Theme 4: Societal Factors

Participant	Code	Transcript Excerpt
P3	Economic policies	"The rising cost of healthy foods compared to junk foods really limits my ability to eat nutritiously."
P7	National nutrition programs	"I've benefited from government assistance programs that provide vouchers for fresh produce."
P10	Societal norms	"There's this pervasive idea that eating healthy is expensive and only for the wealthy, which isn't true."
P15	Public health initiatives	"I've seen more public health campaigns promoting the benefits of fruits and vegetables, which is encouraging."
P19	Economic policies	"Government subsidies for corn and soy contribute to the prevalence of cheap, processed foods."
P23	National nutrition programs	"School meal programs play a crucial role in ensuring that children have access to nutritious meals."
P27	Societal norms	"There's pressure to conform to unhealthy eating habits when socializing with friends or at family gatherings."

P30	Public health initiatives	"Health education campaigns in schools have taught me the importance of making nutritious food choices."
P34	Economic policies	"The lack of regulations on food advertising targeting children is concerning and contributes to unhealthy eating habits."
P5	National nutrition programs	"Government-funded nutrition education programs have been instrumental in teaching me how to make healthier food choices."
P12	Societal norms	"Cultural traditions often revolve around high-calorie, high-fat foods, making it challenging to adopt healthier eating habits."
P17	Public health initiatives	"Public health initiatives like soda taxes and menu labeling have made me more mindful of my food choices."
P21	Economic policies	"Low wages and unstable employment make it difficult to afford nutritious foods, even when I want to eat healthily."
P25	National nutrition programs	"The WIC program provides essential support for low-income families like mine to access nutritious foods."
P28	Societal norms	"There's a stigma attached to eating healthy foods, with some people equating it with being elitist or pretentious."
P32	Public health initiatives	"Community health fairs and screenings have raised awareness about the importance of a balanced diet."
P35	Economic policies	"Tax breaks for agribusinesses incentivize the production of unhealthy processed foods over fresh produce."
P8	National nutrition programs	"Government-funded school breakfast programs ensure that children start the day with a nutritious meal."
P14	Societal norms	"Social media influencers often promote fad diets and unhealthy eating habits, which can be misleading."
P20	Public health initiatives	"The '5-a-Day' campaign has been effective in encouraging me to eat more fruits and vegetables."
	**Theme 5: Intersectionality**	

Participant	Code	Transcript Excerpt
P3	Intersectional identities	"As a single parent working multiple jobs, finding time and resources for nutritious meals can be a challenge."
P7	Complexity of factors	"My cultural background influences my food preferences, but so does my socioeconomic status and access to affordable options."
P10	Interconnected influences	"It's not just about personal choices; systemic barriers and discrimination play a significant role in shaping my diet."
P15	Cumulative impact	"The intersection of race, income, and neighborhood environment creates layers of challenges in accessing healthy foods."
P19	Interplay of identities and systems	"Being a member of a marginalized community means facing barriers at multiple levels when it comes to food access."
P23	Overlapping inequalities	"Being low-income and living in a food desert amplifies the challenges of maintaining a nutritious diet."
P27	Intersectional barriers	"Gender norms and expectations affect my food choices and access to nutritious meals in ways that aren't always obvious."
P30	Systemic inequalities	"Structural racism and economic disparities contribute to disparities in food access and health outcomes."
P34	Cumulative disadvantages	"Growing up in poverty has lifelong effects on diet and health, compounded by other factors like ethnicity and education."
P5	Interconnected influences	"My disability affects my ability to prepare meals and access certain foods, intersecting with other factors like income."
P12	Intersectional identities	"Being a member of the LGBTQ+ community comes with its own set of challenges when it comes to accessing culturally appropriate foods."
P17	Complexity of factors	"My immigrant background shapes my food preferences, but so does my education level and social support network."

P21	Interplay of identities and systems	"Being a racial minority and living in a low-income neighborhood compounds the barriers to accessing healthy foods."
P25	Overlapping inequalities	"Gender roles within my household affect who is responsible for meal preparation and grocery shopping, influencing our diet."
P28	Intersectional barriers	"As a person with a chronic illness, dietary restrictions intersect with financial constraints and limited food options."
P32	Systemic inequalities	"Discrimination in the healthcare system affects the quality of nutrition education and resources available to marginalized groups."
P35	Cumulative disadvantages	"The intersection of disability, race, and socioeconomic status creates unique challenges in accessing nutritious foods."
P8	Interconnected influences	"My cultural heritage influences my food choices, but so does my level of education and income."
P14	Complexity of factors	"Being a caregiver for elderly parents adds another layer of complexity to managing our household's dietary needs."
P20	Interplay of identities and systems	"Living in a rural area means limited access to fresh produce, compounded by transportation barriers and income constraints."
